# Attitudes toward concordance in psychiatry: a comparative, cross-sectional study of psychiatric patients and mental health professionals

**DOI:** 10.1186/1471-244X-12-53

**Published:** 2012-05-30

**Authors:** Carlos De las Cuevas, Amado Rivero-Santana, Lilisbeth Perestelo-Pérez, Jeanette Pérez-Ramos, Pedro Serrano-Aguilar

**Affiliations:** 1 University of La Laguna, Faculty of Medicine, Campus de Ofra s/n, San Cristóbal de La Laguna, Canary Islands 38071, Spain; 2 CIBER de Epidemiología y Salud Pública (CIBERESP), Barcelona, Spain; 3 Fundación Canaria de Investigación en Salud (FUNCIS), Santa Cruz de Tenerife, Spain; 4 Health Technology Assessment Unit, Canary Islands Health Service, Government of the Canary Islands, Pérez de Rozas 5, 4th floor, Santa Cruz de Tenerife, Canary Islands 38004, Spain

**Keywords:** Attitudes, Concordance, LATCon, Psychiatric outpatients, Psychiatrist, Psychiatry registrar, Shared decision-making

## Abstract

**Background:**

Concordance and Shared Decision-Making (SDM) are considered measures of the quality of care that improves communication, promotes patient participation, creates a positive relationship with the healthcare professional, and results in greater adherence with the treatment plan.

****Methods**:**

This study compares the attitudes of 225 mental health professionals (125 psychiatrists and 100 psychiatry registrars) and 449 psychiatric outpatients towards SDM and concordance in medicine taking by using the "Leeds Attitude toward Concordance Scale" (LATCon).

**Results:**

The internal consistency of the scale was good in all three samples (Cronbach's α: patients = 0.82, psychiatrists = 0.76, and registrars = 0.82). Patients scored significantly lower (1.96 ± 0.48) than professionals (*P* < .001 in both cases), while no statistically significant differences between psychiatrists (2.32 ± 0.32) and registrars (2.23 ± 0.35) were registered; the three groups showed a positive attitude towards concordance in most indicators. Patients are clearly in favor of being informed and that their views and preferences be taken into account during the decision-making process, although they widely consider that the final decision must be the doctor's responsibility. Among mental health professionals, the broader experience provides a greater conviction of the importance of the patient's decision about treatment.

**Conclusions:**

We observed a positive attitude towards concordance in the field of psychotropic drugs prescription both in professionals and among patients, but further studies are needed to address the extent to which this apparently accepted model is reflected in the daily practice of mental health professionals.

## Background

In the last few decades there has been an increasing interest in the involvement of patients in their treatment decisions [1]. The so called "disease-centered model" in healthcare, where physicians make treatment decisions based on their technical knowledge and clinical data, has evolved towards a ”patient-centered model”, where patients become active participants in their own care and their individual needs and preferences are taken into account when making decisions about their treatment or diagnostic procedures [1-4]. Patient-centered care is currently recognized as a measure of the quality of healthcare [1]. Studies report that patient-centered care improves communication, promotes patient involvement in care, creates a positive relationship with the provider, and results in improved adherence with the treatment plan [5,6].

Concerning drug prescription, this new paradigm has led to the introduction of the concept of "concordance" defined as "… an agreement between patient and health professional reached after a negotiation that respects the patient's beliefs and wishes to determine whether they want to be treated, when and how to take their medication, and recognizing that the patient's decision is the most important " [7]. In contrast with traditional concepts such as "adherence" or "compliance" that reflect a paternalistic conception of the doctor-patient relationship based on a medical model of disease, concordance not only refers to the patient’s medication-taking behavior but rather the nature of the interaction between therapist and patient, based on the notion that consultations between clinicians and patients are a negotiation between equals, which aims to establish a therapeutic alliance [8,9]. In an attempt to operationalize this new construct, Raynor et al. [10] developed the scale "Leeds Attitude toward Concordance", a questionnaire designed to assess the attitudes of health professionals towards the concept of concordance, later adapted for use in patients [11].

Concordance is closely related to the more widespread concept of shared decision-making (SDM). This is a patient-centered approach in which the healthcare professional and patient exchange information on the best available treatment and discuss the implications of each option [12-14]. In the process, patient autonomy is respected, the patient is assisted with setting their values and preferences, and final treatment decisions reflect a mutual agreement between patient and physician rather than a unilateral decision taken solely by the physician. In this sense, concordance can be considered as the desired outcome of a SDM process [15], but it also has been conceptualized as a subset of SDM limited to the prescription of medications [16].

Trevena and Barratt [17] consider that the suitability of a decision in SDM depends on the clinical context, patient preferences, and the responsibility of healthcare professionals. However, not all patients are prepared, suitable, or want to participate to the same degree in the process of making decisions about the treatment of their disease. Some may want to play an active role in discussing treatment options, but ultimately want their doctors to be the ones who make decisions on their behalf. For this reason, healthcare professionals and health organizations should not assume that patients want to participate in clinical decision-making, but must assess each patient's preferences and tailor care accordingly.

Research on SDM has concentrated on the field of physical disorders, often in primary care [18,19]. SDM in patients with mental illness has received less attention, and studies are still rare as well as heterogeneous in their designs [20,21]. Studies reveal that most psychiatric patients want to be informed about their treatments and consequences, and that their preferences and values should be taken into account in the decision-making process about their treatments [22-26]. As regards mental healthcare professionals, available results also show a favorable attitude towards SDM, although with some nuances regarding patient characteristics (for example, a possible inability to make decisions) or the specific topic to which the decision relates (e.g., admissions). Intervention studies have applied SDM programs with the aim of improving concordance with psychiatric medications [27-29]. Numerous recent articles have highlighted the urgent need for more research in this area [30-32].

The aim of this paper is to compare the attitudes of mental healthcare professionals and psychiatric outpatients towards SDM and concordance in medicine-taking. Secondly, we want to explore the relationships of these attitudes to biological sex, age, and in the case of mental health professionals, certain variables related to their work. Attending to previous results, we expect that attitudes to concordance were positive in both groups of participants. We are not aware of studies that have analyzed the influence of work experience in mental heath care on attitudes to concordance or SDM; on a speculative basis and taking into account that the patient-centered approach is considered a measure of quality of care, we expect that more experienced psychiatrists were more open to a patient-centered approach.

## Methods

### Samples

Two different samples were recruited for this study. During the first quarter of 2010, 449 consecutive psychiatric outpatients continuously followed for at least one year in two Community Mental Health Centers in Tenerife (Canary Islands, Spain) were invited to participate in the study. Each participant received a full explanation of the study, after which they signed an informed consent document approved by the local ethics committee. Each patient anonymously completed a questionnaire that included socio-demographic and clinical variables and the "Leeds Attitude toward Concordance Scale" (LATCon) while waiting to be seen by their psychiatrists.

An opportunistic sample of mental health professionals attending the XIV Spanish Psychiatry National Congress at Barcelona, Spain, in October 2010, was also obtained. Participants were psychiatrists (n = 125) and psychiatry registrars (n = 100), all of whom had experience of working with psychiatric patients. A psychiatry registrar or resident in psychiatry is a person who has received a medical degree and who practices psychiatry under the supervision of fully licensed psychiatrists during a period of 4 years. Mental health professionals completed the LATCon scale and a questionnaire about socio-demographic and professional information that included: age, biological sex, whether the mental health care was inpatient (hospitalization) or outpatient, and in the case of psychiatrists, academic responsibilities (yes/no), private practice (yes/no), and years of practice in these activities.

### Instrument

The Leeds Attitude toward Concordance scale (LATCon) is a 12-item self-report scale, developed by Raynor et al. (10) that assessed patients’ and health professionals’ attitudes towards concordance in medicine-taking. The respondent scores each item on a four point Likert scale: strongly disagree (0), disagree (1), agree (2) or strongly agree (3). The higher the score in the scale, the more positive the respondent's attitude towards concordance. To facilitate interpretation, the total score is divided by the number of items leading to an average score per item. Raynor et al. (10) reported a reliability of 0.79 (Cronbach's α). This instrument has been translated into Spanish and validated in 435 patients from this study sample (29) showing a monofactorial solution with good reliability (Cronbach's α = 0.82). In order to specifically assess the agreement in a psychiatric context, the term "doctor" was replaced by "psychiatrist".

### Statistical analyses

Age, means, and sex distributions were compared between subsamples using ANOVA and χ^2^ tests, respectively. The differences between groups in their scores on the LATCon were assessed either at scale or at items level by means of ANOVA and MANOVA, respectively. The association between patients’ educational level and LATCon scores was analyzed by means of ANOVA. To explore the relationships between LATCon scores and variables related to the professional background of mental health specialists, the following analyses were performed: in the mental health professionals’ sample (psychiatrists and psychiatry registrars taken together) differences between those working in hospitals and those working in outpatient departments were assessed using a Student *t* test. Later, differences between those with and without academic activity and between those with and without private psychiatric practice were analyzed only in the psychiatrists’ sample (psychiatry registrars are not legally allowed to exercise academic or private practice), using Student t tests. All the analyses were performed using the Statistical Package for the Social Sciences (Version 15; SPSS Inc., Chicago, IL, USA).

## Results

### Sociodemographic and work-related variables

A comparison of socio-demographic variables between the three samples considered is presented in Table 1.

**Table 1:** Sociodemographic characteristics of the participants

**LATCon Items**	**Patients (N = 449)**	**Psychiatrists (N = 125)**	**Psychiatry Registrars (N = 100)**	***P*values**^**a**^
Mean age (SD)	42.7 (11.8)	42.6 (10.3)	28.3 (3.0)	*P* < .001
Female (%)	65	43	56	*P* < .001
No formal education	9.6			
Primary education	44.8			*-*
Secondary education	31.2	-	-	
University degree	14.5			
Inpatient/outpatient units (%)^b^	-	37/63	44/56	*P* = .338
Academic practice (%)	NA	36	NA	-
Private practice (%)	NA	45.6	NA	-

^a^ χ^2^ test, except for age (ANOVA).

^b^ All patients were treated in outpatients units. Statistic test compares psychiatrists and psychiatry registrars.

NA: not applicable.

### Differences at total scale level

The internal consistency of the scale was good for all three samples (Cronbach's α values of 0.82, 0.76, and 0.82 for patients, psychiatrists, and registrars, respectively). Patients’ educational level did not relate significantly to their LATCon scores. Age was not significantly related to the attitude towards concordance in any of the three groups. We performed a two-way ANOVA with sample type and biological sex as independent variables, and the LATCon mean item score as the dependent variable; this yielded a statistically significant effect of sample factor (F(2,659) = 43.4, *P* < .001), indicating a significant difference between groups. Multiple comparisons revealed that patients’ scores (1.96 ± 0.48) were significantly lower than the obtained by mental health professionals (2.32 ± 0.32 for psychiatrists and 2.23 ± 0.35 for psychiatry registrars, *P* < .001 in both cases), whereas no statistically significant differences between the two groups of professionals were recorded. The sample x sex interaction effect was significant only at a 0.10 level (F(2,659) = 3.06, *P* < .064). The student *t* test was used to compare scores from men and women for each of the separate samples (using the Bonferroni correction for Type I error value: 0.05/3 = 0.017); this only yielded a statistically significant difference in the psychiatry registrars sample (t(98) = 2.95, *P* = .004), where women scored lower than men (2.14 ± 0.28 vs. 2.35 ± 0.38).

Taking the two groups of mental health professionals together, those active in hospitalization units showed lower LATCon scores than those who work in outpatient departments (2.23 ± 0.33 vs. 2.32 ± 0.34, respectively) but this difference did not reached statistical significance (t(223) = 1.91, *P* = .057). In the psychiatrists sample, there were not significant differences in attitude towards concordance between those who exercise academic activities and those who do not (2.34 ± 0.41 vs. 2.32 ± 0.27, respectively; t(123) = 0.33, P = .745), nor between those who have a private practice and those who do not (2.33 ± 0.38 vs. 2.31 ± 0.27, respectively; t(123) = 0.34, P = .736). Nor did we obtain any statistically significant relationships between LATCon scores and the number of years of academic work (N = 46; Pearson r = 0.12, P = .420) or private practice (N = 57; Pearson r = 0.18, P = .163). For psychiatry registrars, the number of years of residence does not correlate significantly with the attitude to concordance (N = 100; Pearson r = 0.17, P = .099).

### Differences at Items level

Table 2 shows the mean values, standard deviations, and percentage of participants who agreed or strongly agreed with each scale item in the samples studied, and the results of a MANOVA with the sample type as the independent variable and the 12 LATCon items as dependent variables. The results obtained are in line with the global scale; the majority of items show values above 1.5 (i.e., in general the three groups are in favor of the concordance on most indicators). However, patients scored significantly lower than mental health professionals in all except two items (items 1 and 4), with item 10 ("During the psychiatrist-patient consultation, it is the patient’s decision that is most important”) showing the higher disagreement (mean score 1.12 and 75 % of them expressed strongly disagree or disagree). Psychiatrists and psychiatry registrars only differed significantly for item 10 with the psychiatrists registering the highest scores. This item and item 1 (“The consultation between the psychiatrist and patient should be viewed as a negotiation between equals”) were those which registered greater disagreement among mental health professionals.

**Table 2:** Means (standard deviation), percentage of participants agreeing or totally agreeing, and MANOVA results

**LATCon Items**	**Patients**		**Psychiatrists**		**Psychiatry Registrars**		***P*values**
1. The consultation between the psychiatrist and patient should be viewed as a negotiation between equals	1.7 (1.0)	61 %	1.5 (0.8)	51 %	1.7 (0.7)	67 %	*P* = .16
2. Psychiatrists should respect their patients’ personal beliefs and how they cope	2.3 (0.7)	91 %	2.7 (0.5)	99 %	2.7 (0.5)	99 %	*P* < .001; Pat < Psy, Reg
3. The best use of medicine is when it is what the patient wants and is able to achieve	1.9 (0.8)	78 %	2.4 (0.6)	96 %	2.3 (0.6)	92 %	*P* < .001; Pat < Psy, Reg
4. Just as prescribing is an experiment performed by the psychiatrist, so too is medication taking an experiment performed by the patient	1.4 (1.0)	48 %	1.3 (0.9)	46 %	1.4 (0.9)	43 %	*P* = .65
5. Psychiatrists should give patients the opportunity to talk about their thoughts about their illness and negotiate how it is treated	2.1 (0.9)	78 %	2.5 (0.6)	96 %	2.4 (0.6)	95 %	*P* < .001; Pat < Psy, Reg
6. Better health would follow from cooperation between psychiatrists and patients	2.5 (0.7)	95 %	2.9 (0.3)	100 %	2.8 (0.4)	100 %	*P* < .001; Pat < Psy, Reg
7. A high priority in the consultation between psychiatrist and patients is to establish agreement about the need for medicine	2.1 (0.9)	83 %	2.6 (0.5)	99 %	2.5 (0.6)	95 %	*P* < .001; Pat < Psy, Reg
8. Psychiatrists should be sensitive to patient desires, needs and abilities	2.2 (0.8)	88 %	2.7 (0.5)	99 %	2.5 (0.5)	99 %	*P* < .001; Pat < Psy, Reg
9. Psychiatrists should try to help patients to make as informed a choice as possible about the benefits and risks of alternative treatments	2.4 (0.7)	92 %	2.6 (0.5)	98 %	2.6 (0.5)	99 %	*P* < .001; Pat < Psy, Reg
10. During the psychiatrist-patient consultation, it is the patient’s decision that is most important	1.1 (0.9)	25 %	1.7 (0.8)	54 %	1.4 (0.7)	35 %	*P* < .001; Pat < Reg < Psy
11. Psychiatrists should be more sensitive to how patients react to the information they give	2.0 (0.8)	80 %	2.5 (0.5)	100 %	2.3 (0.5)	97 %	*P* < .001; Pat < Psy, Reg
12. Psychiatrists should try to learn about the beliefs their patients hold about their medicines	1.8 (0.9)	71 %	2.4 (0.6)	95 %	2.4 (0.6)	97 %	*P* < .001; Pat < Psy, Reg

Abbreviations: Pat, Patients; Psy, Psychiatrists; Reg, Psychiatry Registrars.

## Discussion

Our results show that both psychiatric outpatients and mental health professionals are in favor of the concept of concordance, a result consistent with those obtained in previous studies [22-26]. However, patients scored significantly lower than professionals at both the scale level and in the majority of questionnaire items.

Concerning patients, previous studies have shown that although they are clearly in favor of being informed and that their views and preferences should be taken into account during the decision-making process, when asked who should be responsible for the final decision most of them prefer to delegate to the doctor [33]. This is reflected in the response to the questionnaire item 10 ("During the psychiatrist-patient consultation, it is the patient’s decision that is most important"), in which patients show their higher rate of disagreement, while for psychiatrists, although this item also registers the lowest rates of agreement, the percentage of agreement is more than double that corresponding to patients. This result has two not mutually exclusive implications: first, it suggests that the representation of a paternalistic doctor-patient relationship would be more rooted in the minds of patients than in professionals, contradicting the idea of health professionals as reluctant to lose their dominant role in their interaction with a patient who would prefer greater involvement in decision-making. Second, it suggests the need to develop theoretical models that differentiate between a process of deliberation between doctor and patient (characterized by mutual communication and empathy for the patient's feelings and values) and a final act of determining the decision to make [34,35]. In any case, concerning mental health professionals, we must not rule out a possible effect of social desirability, to the extent that participation and empowerment of the patient are now highly valued in the conceptualization of healthcare.

The only statistically significant difference between psychiatrists and psychiatry registrars was obtained in the aforementioned item 10, with registrars showing lower scores. It is possible to hypothesize that the wider experience of psychiatrists has provided them with a greater conviction of the importance of the patient's decision about treatment, perhaps in relation to medication adherence. However, none of the professionals variables assessed was significantly related to the attitude towards concordance.

Within the group of psychiatry registrars, women scored significantly lower on the total scale and it is not easy to find explanations for this result, especially when studies on the facilitation of SDM in consultation by the doctor found no statistically significant differences between the sexes [36,37]. In addition, the literature on doctor-patient communication shows women as more empathic and involved in collaborative behaviors and discussion of psychosocial topics [38,39]. However, a recent study in the context of SDM in primary care reveals that being female and registrar status are significant predictors of anxiety about the uncertainty in the treatment process [40], which could impair professional involvement in SDM. Finally, we obtained a difference with tendency towards statistical significance between those who work in hospitalization units compared to those who work in outpatient departments (lower scores in the former). This result could be accounted for because patients admitted usually have more serious conditions, which would imply a decline in the patient's decisional capacity, whether real or perceived by professionals.

From a psychometric point of view, there is a strong ceiling effect on the scores of the participants, especially in samples of professionals. This low variability may be responsible for the absence of significant relationships between attitude towards concordance and other variables considered. Future studies would analyze the functioning of LATCon scale compared to other measures of attitudes toward SDM, and explore the possibility of assessing such attitudes from a less abstract level, in order to identify more specific components in which more intense individual differences could be observed.

This study represents an initial attempt to analyze differences in attitudes to concordance between psychiatric patients and professionals in Spain, and it presents several limitations. First, samples may not be representative of Spanish psychiatric patients and professionals, especially this latter sample that was recruited at a psychiatry congress. Second, patients’ diagnoses were not collected because of confidentially issues, and therefore, responses between different diagnostic categories could not be compared. However, previous studies that include patients with different mental disorders did not obtain statistically significant differences between diagnostic categories [22,41], and mean scores on the Autonomy Preference Index (API - decision-making subscale) are quite similar across two studies that respectively include patients with schizophrenia [20] and depression [42]. Third, potential confounding variables such as social desirability or patients’ trust in the physician were not assessed.

Patient-centered psychiatry advocates a paradigm shift of attention to mental health problems, moving from a focus on diseases, typical of techno-medicine, to the humanization of care that facilitates patient cooperation because it considers the patient as the center of clinical care and considers their values and expectations, allowing the full integration of the psychological, behavioral, and social aspects of illness postulated by Engel's biopsychosocial model [43] based on systems theory. Our study demonstrates a positive attitude towards SDM in the field of prescription of psychotropic drugs in both mental health professionals as well as among psychiatric outpatients, but future studies need to be addressed in order to clarify to what extent this model, although apparently accepted, is always reflected in the daily practice of mental health professionals.

According to a recent systematic review [44], no firm conclusions can be drawn at present about the effects of shared decision-making interventions for people with mental health conditions although there is no evidence of harm to the patients. In our opinion, it is not simply a matter of results, but rather application of the fundamental rights of a group of patients who have not yet sufficiently benefited from the empowerment of consumers in the same way as other fields of medicine. For us, shared decision-making is an ethical and legal imperative of current clinical psychiatric practice.

## Conclusions

A positive attitude towards SDM in the field of psychotropic drugs prescription was observed both in mental health professionals and among psychiatric outpatients, but further studies are needed to address the extent to which this apparently accepted model is reflected in the daily practice of mental health professionals.

## Competing interests

The authors declare that they have no competing interests.

## Authors' contributions

CDC conceived the study, participated in the design and the coordination of the study and drafted the manuscript. ARS performed the statistical analyses and assisted in drafting the manuscript. LPP participated in the design and the coordination of the study. JPR and PSA participated in the analytical plan, the interpretation of the results, and assisted in drafting the manuscript. All authors read and approved the final manuscript.

## Pre-publication history

The pre-publication history for this paper can be accessed here:

http://www.biomedcentral.com/1471-244X/12/53/prepub
